# Development of a Phone Survey Tool to Measure Respectful Maternity Care During Pregnancy and Childbirth in India: Study Protocol

**DOI:** 10.2196/12173

**Published:** 2019-04-25

**Authors:** Amnesty E LeFevre, Kerry Scott, Diwakar Mohan, Neha Shah, Aarushi Bhatnagar, Alain Labrique, Diva Dhar, Sara Chamberlain, Rajani Ved

**Affiliations:** 1 Division of Epidemiology and Biostatistics School of Public Health and Family Medicine University of Cape Town Cape Town South Africa; 2 Department of International Health Johns Hopkins Bloomberg School of Public Health Baltimore, MD United States; 3 Oxford Policy Management New Delhi India; 4 Bill and Melinda Gates Foundation New Delhi India; 5 BBC Media Action Delhi India; 6 National Health Systems Resource Center Delhi India

**Keywords:** maternal care, text messages, phone surveys, India

## Abstract

**Background:**

Respectful maternity care (RMC) is a key barometer of the underlying quality of care women receive during pregnancy and childbirth. Efforts to measure RMC have largely been qualitative, although validated quantitative tools are emerging. Available tools have been limited to the measurement of RMC during childbirth and confined to observational and face-to-face survey modes. Phone surveys are less invasive, low cost, and rapid alternatives to traditional face-to-face methods, yet little is known about their validity and reliability.

**Objective:**

The primary objective of this study was to develop validated face-to-face and phone survey tools for measuring RMC during pregnancy and childbirth for use in India and other low resource settings. The secondary objective was to optimize strategies for improving the delivery of phone surveys for use in measuring RMC.

**Methods:**

To develop face-to-face and phone surveys for measuring RMC, we describe procedures for assessing content, criterion, and construct validity as well as reliability analyses. To optimize the delivery of phone surveys, we outline plans for substudies, which aim to assess the effect of survey modality, and content on survey response, completion, and attrition rates.

**Results:**

Data collection will be carried out in 4 districts of Madhya Pradesh, India, from July 2018 to March 2019.

**Conclusions:**

To our knowledge, this is the first RMC phone survey tool developed for India, which may provide an opportunity for the rapid, routine collection of data essential for improving the quality of care during pregnancy and childbirth. Elsewhere, phone survey tools are emerging; however, efforts to develop these surveys are often not inclusive of rigorous pretesting activities essential for ensuring quality data, including cognitive, reliability, and validity testing. In the absence of these activities, emerging data could overestimate or underestimate the burden of disease and health care practices under assessment. In the context of RMC, poor quality data could have adverse consequences including the *naming and shaming* of providers. By outlining a blueprint of the minimum activities required to generate reliable and valid survey tools, we hope to improve efforts to develop and deploy face-to-face and phone surveys in the health sector.

**International Registered Report Identifier (IRRID):**

DERR1-10.2196/12173

## Introduction

### Background and Rationale

In 2015, nearly 90% of the estimated 302,000 global maternal deaths occurred in 2 regions: sub-Saharan Africa (201,000) and Southern Asia (66,000) [1]. Although the global number of maternal deaths in 2015 corresponds to an absolute decline in the maternal mortality ratio (MMR) of 44% since 1990, it too masks wide variation within and across countries as nearly 30% of countries assessed globally have not achieved significant declines in MMR [1]. Historically, efforts to achieve reductions in mortality have sought to bolster the frequency and timeliness of health service utilization across the continuum of care, with particular emphasis on pregnancy care and institutional delivery. Although this has led to increases in the overall utilization of care in many settings [2,3], the lack of momentum in realizing declines in maternal mortality raises important questions about the underlying quality of care received during pregnancy and childbirth.

The treatment of women during childbirth has emerged as a key component of overall quality of care. Building off of research on obstetric violence from Latin America, the closely related term *disrespect and abuse* has been used in recent years to describe varying typologies of the mistreatment of women during childbirth [4,5]. Emerging evidence on disrespect and abuse suggests that poor treatment of women during childbirth may be widespread and a barrier to improving maternal health outcomes and continued engagement with the health sector [6].

Ensuing calls to action have framed the mistreatment of women as a violation of human rights and emphasized the right of every woman to respectful maternity care (RMC) [5]. In 2014, the World Health Organization (WHO) issued a statement advocating for the prevention and elimination of disrespect and abuse during facility-based childbirth, stating that “every woman has the right to the highest attainable standard of health, which includes the right to dignified, respectful health care throughout pregnancy and childbirth, as well as the right to be free from violence and discrimination” [7]. In 2016, WHO issued new global guidelines on antenatal care (ANC) during pregnancy [8] as well as standards for improving the quality of maternal and newborn care in health facilities [9] both of which have adopted a human rights–based approach in prioritizing person-centered health and well-being, including the provision and experience of care.

### Innovations in the Measurement of Disrespect and Abuse

Increased attention to RMC, coupled with country-level efforts to implement new guidelines for ANC [8], presents a unique opportunity to bolster efforts to measure women’s experiences with facility-based services during pregnancy and childbirth, including disrespect and abuse. To date, efforts to measure disrespect and abuse have largely employed qualitative methods and focused primarily on childbirth at the exclusion of understanding probable linkages with care received during pregnancy. A body of work is emerging, which aims to develop validated quantitative survey tools for the measurement of RMC through direct observation and/or structured face-to-face surveys [10-12]. Findings from a recent systematic review have identified and presented validated instruments for measuring women’s childbirth experiences [11]. Although this study helps to synthesize the state of current tools, including their dimensions, response options, and psychometric properties [13], additional research is needed to refine the optimal content, timing, and location of survey implementation. Furthermore, in light of the intensive resource requirements associated with direct observations and face-to-face surveys, low-cost alternative survey modalities are needed, which could allow for the routine, rapid, and real-time measurement of women’s pregnancy and childbirth experiences, including disrespect and abuse.

Near ubiquitous access to mobile phones globally has catalyzed discourse on the potential of phone surveys for use in the monitoring of population health. Although gender gaps in mobile phone access [14], coupled with uncertain digital literacy, raise important questions about the reliability and validity of phone surveys, they nevertheless may serve as a low-cost, minimally invasive, rapid means of data gathering. In contrast to resource and time-intensive face-to-face surveys, phone surveys offer respondents the option of being interviewed over a personal or shared mobile phone in the privacy of their own home through one of several modalities: Unstructured Supplementary Services Data (USSD), short message service (SMS), interactive voice response (IVR), and computer-assisted telephone interview (CATI) survey modalities [15,16]. In USSD and SMS surveys, respondents answer questions via text message, whereas in IVR surveys, users listen to automated prerecorded voice prompts, which include multiple choice questions and preset answers. The respondent selects the answer by pressing a corresponding number on the keypad or touch-tone phone (eg, “Press 1 for English, 2 for Hindi”). In contrast, CATI surveys employ human interviewers to implement the survey using a script and data capture tool, which could be paper- or software-based [16].

A recent systematic review identified 19 applications of phone surveys in low- and middle-income countries (LMICs) employing varying modalities including 10 CATI, 6 IVR, and 3 SMS surveys [17]. Survey locations have been diverse (South Asia, Latin America, and Africa) and covered a range of topics on health and socioeconomics, including assets, employment, and food security [17]. Participant recruitment has predominately relied on household baseline surveys to collect mobile phone numbers [17]. Less common were alternatives such as Random Digit Dialing (RDD) or phone numbers drawn from mobile network operators [17]. Overall findings from phone surveys conducted to date suggest that the modality of survey implementation is a critical consideration affecting cost, survey metrics (including length and response options), and quality [17]. CATI surveys, although costlier because of their human resource requirements, resulted in higher response and completion rates [17]. The further implementation through human contact, which permits personalized responses to clarify questions, may additionally translate to improved data quality and lower attrition [17].

In response to calls to improve the standardization of phone survey assessments, research is emerging, which proposes to systematically test the effects of alternative survey modalities on factors influencing cost and key survey metrics, including contact, response, completion, and refusal rates as well as demographic representativeness [18]. Although this body of research is promising, details remain outstanding on the procedures undertaken for validating the survey tools implemented through the phone survey modalities and, in particular, on the assurances that quality criteria are met [13]. Even in instances where validated face-to-face survey tools are utilized as the basis for the phone survey tool, modifications to survey formats, including length and response options and enumerator gender as well as incentives, may be required, which could influence data quality and survey findings. The further influence of the underlying sampling frame from which phone numbers are drawn on data quality and generalizability may also influence findings, particularly in instances where face-to-face population-based surveys are not used to facilitate initial recruitment/participation. Collectively, these factors reiterate the importance of evaluating quality criteria in the development of phone surveys.

In this protocol study, we outline research underway in India to develop validated phone survey tools appropriate for use in the routine measurement of RMC during pregnancy and childbirth in India. Although concurrent efforts are underway as part of the same study to develop phone survey tools for measuring satisfaction and motivation among Accredited Social Health Activists, as well as essential newborn care and infant feeding practices, processes will mirror those proposed for RMC. Study activities will draw from a population-based sample of pregnant and postpartum women with access to mobile phones in 4 districts of Madhya Pradesh (MP). Research activities include substudies on (1) cognitive testing to assess face validity and optimize phone survey tool content; (2) test-retest to determine the reliability of the face-to-face survey modality; and (3) CATI versus face-to-face surveys (intermodal reliability) [13]. To optimize the delivery of phone surveys, we outline plans for analyses exploring the effects of content on survey response, completion, and attrition rates. Research findings are anticipated to result in the development of a valid and reliable phone survey tool for the routine measurement of RMC during pregnancy and childbirth in India.

## Methods

### Study Setting

Data collection is part of the impact evaluation of Kilkari; an IVR-based maternal messaging program that aims to empower women through improved access to essential health information. Led by the Ministry of Health and Family Welfare (MOHFW) and implemented by BBC Media Action with support from the Bill & Melinda Gates Foundation, United States Agency for International Development, and the Barr Foundation, Kilkari provides weekly stage-based audio messages on topics including birth preparedness, family planning, and maternal and child nutrition directly to the mobile phones of pregnant and postpartum women up to 1 year postpartum. With implementation currently underway in 13 states across India, Kilkari has delivered prerecorded audio content to 8.3 million users in 33 months [19].

Data collection is occurring in 4 districts (Mandsaur, Hoshangabad, Rewa, and Rajgarh) of MP. MP is located in the geographic heart of India and is home to a population of over 75 million. Among women, an estimated 59% are literate (as compared with 82% of men), 64% have ever attended school, and 29% have access to a mobile phone [3]. In 2015, 53% of pregnant women attended ANC in the first trimester, 36% received the recommended 4 ANC visits, 81% delivered in a health facility, 78% had births attended by a skilled provider, and 18% received a postnatal health check within 2 days following birth [3]. Data on differentials in health outcomes and/or utilization of health services among those with and without access to mobile phones are not available.

Across all 4 districts in MP, data collection is occurring among a subsample of pregnant and postpartum women identified as part of a household listing exercise. During the household listing, all women of reproductive age with access to a mobile phone are identified. Women who are 4 to 7 months pregnant as well as those with a reported pregnancy outcome in preceding 1 to 4 months are then interviewed as part of the pregnant and postpartum women’s surveys.

### Measuring Respectful Maternity Care

Freedman and Kruk define disrespect and abuse during childbirth as “interactions or facility conditions that local consensus deems to be humiliating or undignified, and those interactions or conditions that are experienced as or intended to be humiliating or undignified” [20]. Building off of this definition and a 2010 landscape analysis by Bowser and Hill [21], Bohren et al outlined 7 categories of disrespectful and abusive care during childbirth: (1) physical abuse; (2) sexual abuse; (3) verbal abuse; (4) stigma and discrimination; (5) failure to meet professional standards of care; (6) poor rapport between women and providers; and (7) health system conditions and constraints [6]. These categories were subsequently conceptualized in 2 dimensions: (1) intentional use of violence, including physical abuse, verbal abuse, and negligent withholding of care and (2) structural disrespect, which stems from “deviations from accepted standards for infrastructure, staffing, equipment availability, and supplies needed to deliver care, as well as in unnecessary interventions, demands for illegal payments, and the detainment of people in facilities until they have paid their bills” [22].

**Figure 1:**
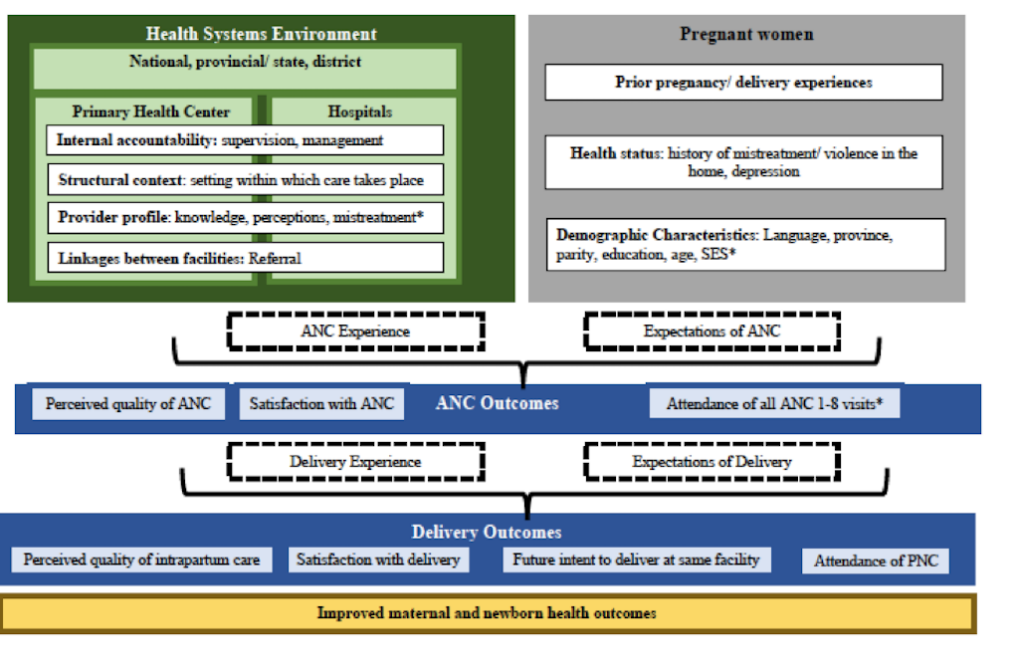
Conceptual framework for measuring respectful maternity care (RMC).

In this protocol study, we focus on the measurement of each of these major typologies of disrespect and abuse along with the underlying contextual factors that underpin them. Figure 1 outlines a conceptual framework for measuring RMC during pregnancy and intrapartum care, which brings together traditional approaches to measuring quality of care [9,23-27] with frameworks for assessing mistreatment of care during childbirth [28]. Viewing mistreatment through the lens of one perspective (eg, intrapartum women) at a single time point (eg, childbirth), although important, may nevertheless provide a limited view of the larger context within which treatment occurs and the risk factors underpinning it. This framework aims to illustrate that maternal health outcomes stem from the interaction of beneficiaries with providers in a complex and evolving community and health systems context through multiple points of contact in different facilities starting with ANC in primary health centers. We posit that women’s interactions with the larger health systems’ environment help to formulate their care experience and expectations, and ultimately outcomes, including utilization of services and perceptions of quality and satisfaction.

Multimedia Appendix 1 summarizes questions by RMC typology and domain for the proposed measurement of RMC during childbirth. Table 1 summarizes the number of questions by RMC domain for each of the survey planned and compares these against alternatives in the literature. In contrast to approaches in Kenya, Bihar, and Ethiopia, we distinguish questions in MP according to whether they aim to estimate the prevalence of a particular domain or rather users’ satisfaction with an aspect of care received. This distinction is important given its implications on the response options required (eg, Likert scales versus binary or categorical) and their associated implications for analyses. Measurement of RMC will occur through 2 modalities: (1) face-to-face survey and (2) phone surveys. Face-to-face surveys will be carried out on 2 populations as part of a larger baseline evaluation of Kilkari: (1) women who are 5 to 7 months pregnant and (2) women with a birth outcome in the preceding 1 to 4 months. In addition to RMC, face-to-face surveys include modules on mobile access and literacy, socioeconomic and demographic characteristics, birth history, and experiences with care during pregnancy or childbirth. Face-to-face surveys will be modified following analyses to yield the following phone survey tools: (1) RMC during pregnancy; (2) RMC during childbirth; and (3) essential newborn care and infant feeding.

**Table 1:** Comparison and summary of total number of questions by respectful maternity care domain for Madhya Pradesh, India, and other respectful maternity care studies identified in the literature.

Domains		Afulani et ala PCMC in Kenya [12]		Bihar India [29]		Sherferaw et al Ethiopia [10]		Madhya Pradesh India			
								Prevalence module		Satisfaction module	
		No.	Response options	No.	Response options	No.	Response options	No.	Response options	No.	Response options
**Physical or sexual abuse**											
	Use of force	1	Likert scale 1-5	3	Binary; Categorical	2	Likert scale 1-5	2	Binary, Categorical	—	—
	Physical restraint	—^a^	—	—	—	—	—	—	—	—	—
**Verbal Abuse**											
	Harsh or rude language	1	Likert scale 1-5	2	Binary; Categorical	3	Likert scale 1-5	2	Binary, Categorical	—	—
	Threats and blaming	—	—	—	—	—	—	—	—	—	—
	Judgmental or accusatory comments	—	—	1	Categorical	—	—	—	—	—	—
**Stigma and discrimination**											
	Discrimination	1	Likert scale 1-5	1	Categorical	4	Likert scale 1-5	2	Categorical	—	—
**Failure to meet professional standards of care**											
	Refusal to provide pain relief	2	Likert scale 1-5	—	—	1	Likert scale 1-5	—	—	1	Likert scale 1-6
	Lack of informed consent process	4	Likert scale 1-5	1	Categorical	3	Likert scale 1-5	3	Binary, Categorical	2	Likert scale 1-6
	Breaches of confidentiality	2	Likert scale 1-5	1	Binary	1	Likert scale 1-5	1	Binary		
	Neglect, abandonment, or long delays	1	Likert scale 1-5	2	Binary; Categorical	3	Likert scale 1-5	2	Binary, Categorical	—	—
	Skilled attendant absent at time of delivery	—	—	—	—	—	—	—	—	—	—
	Painful vaginal exams	—	—	—	—	—	—	—	—	—	—
**Poor rapport between women and providers**											
	Poor communication	6	Likert scale 1-5	1 question 9 subcategories	Binary	4	Likert scale 1-5	1	Binary	2	Likert scale 1-6
	Language and interpretation issues	1	Likert scale 1-5	—		1	Likert scale 1-5	—	—	—	—
	Lack of supportive care from health workers	6	Likert scale 1-5	1	Likert scale 1-5	9	Likert scale 1-5	1	Binary	1	Likert scale 1-6
	Trust	2	Likert scale 1-5	—	—	—	—	—	—	—	—
	Denial or lack of birth companions during labor and delivery	2	Likert scale 1-5	2	Binary, Categorical	1	Likert scale 1-5	4	Binary, Categorical	—	—
	Lack of respect for women’s preferred birth positions/ freedom of movement	—	—	3	Binary	1	Likert scale 1-5	2	Binary	—	—
	Denial of safe traditional practices	—	—	—	—	1	Likert scale 1-5	—	—	—	—
	Detainment in facilities	1	Likert scale 1-5	2	Binary, Continuous			2	Binary, Continuous	—	—
	Objectification of women	—	—	—	—	—	—	—	—	—	—
**Health system conditions and constraints **											
	Lack of privacy	1	Likert scale 1-5	1	Binary	2	Likert scale 1-5	1	Binary	—	—
	Bribery and extortion	1	Likert scale 1-5	—	—	1	Likert scale 1-5	1	Binary	—	—
	Safety	1	Likert scale 1-5	—	—	—	—	—	—	—	—
	Physical condition of facilities	4	Likert scale 1-5	—	—	—	—	2	Binary	1	Likert scale 1-6
	Staffing shortages/constraints	1	Likert scale 1-5	—	—	—	—	—	—	—	—
	Supply constraints	—	—	—	—	—	—	—	—	—	—
	Lack of redress	—	—	—	—	—	—	—	—	—	—
	Unclear fee structures	—	—	—	—	—	—	—	—	—	—
	Unreasonable requests of women by health workers	—	—	—	—	—	—	—	—	—	—

Other questions		—	—	2	Likert scale 1-5	—	—	—	—	3	Likert Scale 1-6
Total		38	—	20	—	37	—	26	—	10	—

^a^Question not included.

### Phase 1. Scale and Survey Development

**Figure 2:**
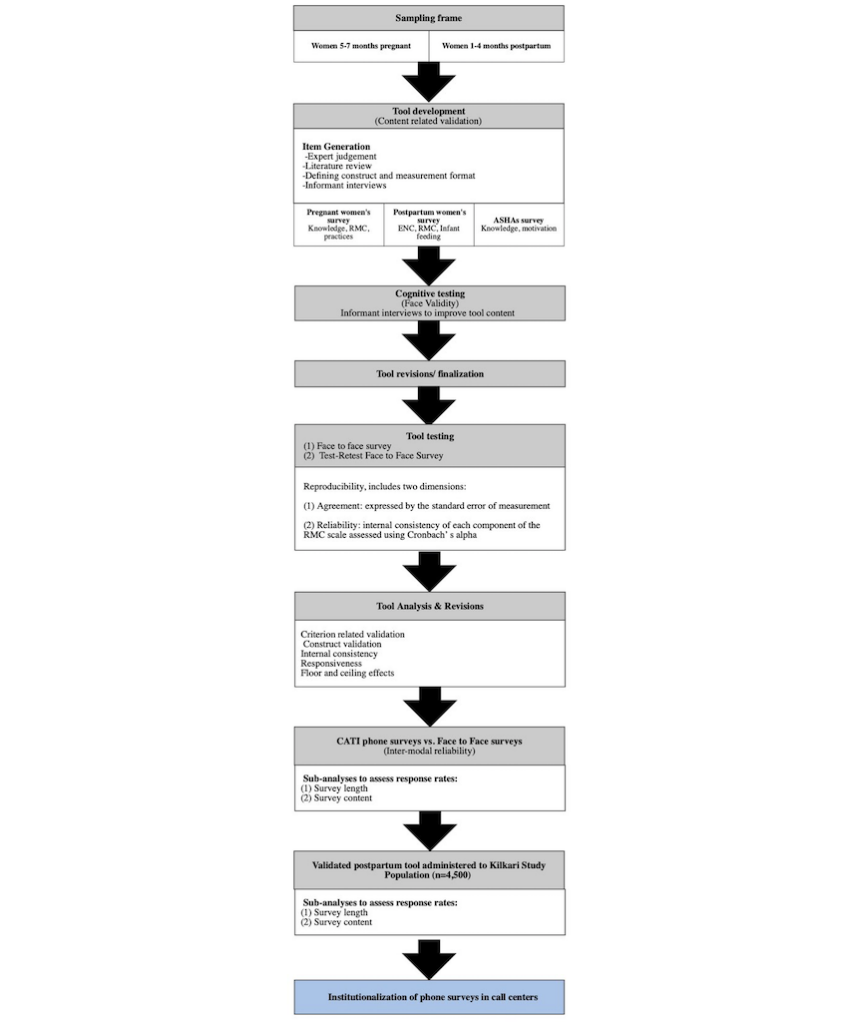
Processes for reliability testing.

Figure 2 outlines proposed processes for validity and reliability testing, whereas Table 2 and Multimedia Appendix 2 summarize survey substudies and validity/reliability tests, respectively. Building off of a strong foundation of existing validated instruments [12], project activities will commence with a literature review from which survey tools will be developed for RMC measurement during pregnancy and childbirth, including scales for measuring satisfaction and prevalence [11]. Item generation for each scale was drawn from concurrent activities underway in Bihar by Rao et al [29] to measure RMC during childbirth through direct observations, exit interviews, and follow-up household interviews during the postpartum period. Indicators from the above listed and other validated survey tools elsewhere in the literature [12] were used in the MP survey tools to allow for cross-site comparison. Once consensus was achieved, items were translated into Hindi and checked by BBC Media Action and MOHFW personnel in Delhi for accessibility, appropriateness of language, tone, and engagingness. Cognitive testing followed in study districts in MP to ensure that survey questions are understandable, appropriate in language and tone, and the words interpreted as intended by varying respondent types.

**Table 2:** Summary description of survey substudies.

Substudy	Objective	Survey activities
Prevalence and scale testing	To determine the prevalence of different typologies of disrespect and abuse	Prevalence surveys in 2 districts of MP, India: RMC^a^ during pregnancy and RMC during childbirth
Reproducibility	To determine the degree to which repeated measurements in stable persons (test-retest) provide similar answers	Test-retest: Face-to-face survey repeated within 14 days
Survey modality	To assess intermodal reliability	Face-to-face survey first, CATI^b^ survey up to 14 days later
Phone survey length and content	To determine the effect of survey length and content on response, completion, and attrition rates	CATI phone surveys: RMC pregnancy phone survey vs RMC childbirth phone survey; Postpartum phone survey
Interrater reliability	To compare demographic characteristics of respondents in larger sampling frame vs those that complete, partially complete, and do not respond to phone surveys	Characteristics of face-to-face survey respondents versus CATI phone survey respondents

^a^RMC: respectful maternity care.

^b^CATI: computer-assisted telephone interview.

### Phase 2. Survey Testing and Refinement

#### Substudy 1: Respectful Maternity Care Scale Testing

Table 3 summarizes the sample size requirements for each substudy. Face-to-face surveys will be conducted to refine the scale and determine the prevalence of different typologies of disrespect and abuse in 4 districts of rural MP. Among pregnant women, a module on RMC during ANC will be integrated into a planned household survey among 5000 women who are 5 to 7 months pregnant. This is sufficient to measure the RMC indicator of reported verbal abuse (assumed 5% prevalence) during pregnancy with 80% power, alpha of .05, and precision of 1%. To measure RMC during childbirth, a total of 880 women with a birth outcome in the preceding 1 to 4 months will be interviewed. This sample size was designed to accommodate survey mode testing described in Phase 3 and is sufficient to additionally measure the prevalence of the RMC indicator of reported verbal abuse (assumed 10% prevalence) during childbirth with 80% power, alpha of .05, and precision of 2%.

Once data are collected, analyses will principally aim to determine the validity of the scale using psychometric analyses (Figure 2). *Criterion-related validity* will be assessed by testing the hypothesis that scale is correlated to measures of reported satisfaction additionally collected as part of the face-to-face survey tool [12,13]. We propose testing this by regressing the main RMC scale and subscales on women’s ratings of their satisfaction with the services and whether they would deliver in the same facility if they were to have another baby [12]. *Construct validity* measures how well the items represent the underlying conceptual structure [13] and will be assessed using factor analysis and the Pearson correlation coefficient between the components. *Reliability analyses* will aim to determine the stability and consistency of results [13]. A Cronbach alpha of .7 or higher is proposed as the cutoff for determining sufficient evidence of reliability [13]. Additional analyses related to the internal consistency of the scale as well as the presence of floor and ceiling effects will be conducted and overall findings on validity and reliability summarized [13].

#### Substudy 2: Reproducibility

To assess reproducibility, a random subsample of pregnant and postpartum women interviewed as part of substudy 1 will be administered a repeat face-to-face survey between 1 and 2 weeks after the initial survey. This substudy will be conducted to determine the degree to which repeated measurements in women interviewed (test-retest) provide similar answers. Assuming a kappa of 0.80, a margin of error of 0.05, an alpha of .05, and the proportion of positive responses of 0.35 for rater 1 and 0.40 for rater 2, 146 participants who have completed the survey are required. Adjusting for a 15% loss to follow-up/refusal between the first and second women’s surveys will require a sample size of 168 women to be interviewed twice. Data will be analyzed for agreement between survey rounds and reliability will be tested using Cohen kappa. The kappa will be adjusted for prevalence and bias, providing Prevalence and Bias Adjusted Kappa.

### Phase 3. Phone Survey Reliability and Delivery Optimization

#### Substudy 3: Survey Mode Testing

Phone survey mode testing will aim to determine the intermodal reliability of face-to-face versus CATI surveys for both the RMC pregnancy and childbirth surveys. Assuming a kappa of 0.80, a margin of error of 0.05, an alpha of .05, and the proportion of positive responses of 0.35 for rater 1 and 0.40 for rater 2, 146 participants who have completed each survey are required. Adjusting for loss to follow-up between the face-to-face women’s survey and the following mobile phone survey, 880 women with a birth outcome in the preceding 1 to 4 months will be interviewed face to face. Within 4 weeks of the initial interview, a random sample of those completing the face-to-face interview who consent to be called for the follow-up phone survey will be contacted. Assuming a 20% response rate, 880 women will be contacted as part of the phone survey to yield the 146 completed face-to-face and phone survey interviews. Only women with access to a mobile phone, aged 18 years or older, and who have had a birth outcome in the preceding 1 to 4 months and are identified in the study districts will be interviewed.

**Table 3:** The number of participants needed by substudy.

Substudy		Study arms	Participants who completed the survey per arm	Total sample size^a^
**ANC^b^** **recipients**				
	Substudy 1: Face-to-face survey of RMC^c^ during ANC	1	400	400
	Substudy 2. Reproducibility (test-retest)	1	168	168
	Substudy 3: Phone survey (intermodal reliability)	1	146	292
	Substudy 4: Interrater reliability	Secondary analysis		
**Intrapartum**				
	Substudy 1: Face-to-face survey of RMC during childbirth	1	400	400
	Substudy 2: Reproducibility (test-retest)	1	168	168
	Substudy 3: Survey mode testing	2	146	292
	Substudy 4: Phone survey length and content	2	294	4500
	Substudy 4: Interrater reliability	Secondary analysis		

^a^The total sample size reflects the sum of the sample across all study arms.

^b^ANC: antenatal care.

^c^RMC: respectful maternity care.

#### Substudy 4: Subanalyses to Optimize Phone Survey Delivery

##### Phone Survey Content and Length

Survey content refers to 2 components of the phone survey: (1) topical area covered and (2) response options and question framing. We will assess the effects on survey content and length (number of questions) of response, completion, and attrition rates using Kaplan-Meier curves to plot survey attrition by time spent for each survey implemented across key populations. This will include comparisons across RMC surveys administered to pregnant and postpartum women. Assuming a baseline survey completion percentage of 20% to detect an absolute 10% difference in survey completion between 2 study arms at an alpha of .05 and power of 80%, it is calculated that 294 individuals who have completed the survey will be needed per study arm. With a completion percentage of 20%, we estimate that 1470 participants would be required. To attain this sample size, the phone survey tool validated in substudy 3 will be applied to the population of 4500 women enrolled in the Kilkari impact evaluation in 4 districts of MP.

##### Interrater Reliability

This subanalysis aims to compare the demographic characteristics of respondents in the larger sampling frame versus those who complete, partially complete, and do not respond to phone surveys. Additional data points, including caste, education, and socioeconomic status, collected during the face-to-face household listing and baseline survey will be juxtaposed against CATI phone survey data.

### Data Management

All data collected will remain in India and will be managed by the India-based research partner. Tablets used for data collection will be password protected. Any adverse events mentioned to the research team during data collection will be brought to the immediate attention of senior project investigators and Institutional Review Boards at Johns Hopkins School of Public Health and in India at Sigma Research and Consulting in New Delhi. Once collected, all data will be deidentified following the merging of data sets as required reliability analyses.

### Ethics Approval

Ethical approval for research activities in India has been obtained from Johns Hopkins School of Public Health’s Institutional Review Board in Baltimore, Maryland, United States, and from Sigma Research and Consulting in New Delhi, India.

## Results

Data collection in India is anticipated to start in July and span through March 2019. Data analyses and report writing will be completed by mid 2019.

## Discussion

### Study Implications

Limited evidence exists on the feasibility of utilizing phone surveys in LMICs for the surveillance of population-level health [17], and no studies to date have been conducted that utilize phone surveys to assess the quality of women’s experiences with care during pregnancy or childbirth in India. Increasing access to mobile phones, particularly in India where a large proportion of maternal and child deaths occur globally, raises the potential for phone surveys to be used in the routine measurement of key health outcomes. Despite their immense potential, the validity and reliability of phone surveys for RMC as compared with traditional face-to-face or direct observations has yet to be determined.

This protocol study aims to catalyze discourse on quality criteria for phone survey validation, which may in part be driven by the survey objectives, the available sampling frame, budget for primary data collection, and context within which data collection is occurring. In many contexts, face-to-face surveys are the starting point for participant recruitment in phone surveys. However, examples of large population-level surveys, which rely on RDD, are emerging [18]. In this protocol study, we consider a sampling frame drawn from population-based recruitment through a face-to-face survey. However, in India, a number of mobile health initiatives, including national-level phone surveys conducted through the Maternal Child Tracking Center call center, draw participants from the phone numbers collected as part of routine health information systems. In light of this, potential future applications at scale of the phone survey tools validated in this study may adopt an RDD approach. Although the population-based recruitment is likely to yield greater population-level representativeness, the sample will still be constrained to women with regular access to a mobile phone. In India, the associated likelihood of selection bias is immense because of differentials in mobile phone access, literacy, and numeracy. Separate analyses planned as part of the Kilkari Impact Evaluation on the intersectionality of ethnicity; gender; education; and phone access, ownership, and use may help to shed light on these differences.

### Rethinking Approaches to Measuring Respectful Maternity Care

To develop validated survey tools, we first conduct a test-retest analysis drawing from survey data collected face-to-face and then conduct interim analyses to refine the tool before administering it over the phone and assessing intermodal reliability. Elsewhere, RMC tools have been developed through direct observations and follow-up face-to-face interviews [30]. Although the direct observations of delivery led to the identification of additional forms of mistreatment, including privacy violations and the failure to ask for consent during vaginal exams [30], they too are not impervious to observer bias in addition to being resource- and time-intensive. In contexts where face-to-face survey tools have been implemented, differences in the typologies of mistreatment have been reported based on the postpartum timing and locale of survey implementation. Findings from a prevalence survey conducted among 1914 women receiving care from a large referral hospital in Dar es Salaam found that 15% of women reported experiencing at least 1 instance of disrespect and abuse during postpartum interviews—a figure that rose to 70% during community follow-up interviews [31]. In this study, we draw from the scale used by Rao et al [29] with the broader aim of allowing for later comparisons with observations and face-to-face survey data collected in Bihar. Although there are contextual differences between MP and Bihar, this may nevertheless allow for additional comparisons to be made.

As part of efforts to validate the survey mode, we have sought to juxtapose face-to-face survey options against CATI surveys. The implementation of CATI surveys is anticipated to differ based on interviewer cadres and available software. In this study, phone surveys will be administered by graduate students identified and supervised by the National Health Systems Resource Centre using tablets containing CAPI survey tools. Future implementation of these surveys once validated is likely to be carried out through national- and/or state-level call centers that may have enumerators with lower levels of education. Care will thus need to be taken to ensure that the tools developed can be easily adopted and administered by enumerators with differing characteristics.

To improve response rates, we have proposed substudies, which aim to optimize phone survey delivery. Limits in resource constraints and the available sample size have meant that we are not testing the effects of introductory language *calls to action* or the incentives (amount, timing, and structure), all of which have been shown to effect response rates. Similarly, we are limited in our ability to assess the effects of the timing of the RMC survey implementation (eg, receiving the survey call immediately after discharge from facility versus several days or weeks later), a factor that may impact response rates and has been shown to influence the reported typologies of disrespect and abuse [31]. In this study, the measurement of RMC during childbirth will occur 1 to 4 months following delivery and thus outside of the health facility environment. Although comparisons of phone survey data from MP, India, will be made for certain items with face-to-face and direct observation data collected in Bihar, differences in the study contexts and populations will limit scope of and conclusions drawn from these analyses.

### Conclusions

This protocol study outlines the proposed strategy for generating validated phone survey tools for the routine, low cost, and rapid measurement of RMC during pregnancy and childbirth in India. Study findings are anticipated to provide a blueprint for the development and validation of phone surveys for the routine measurement of service delivery outcomes in low resource settings.

## Appendix

Multimedia Appendix 1 Draft survey questions for measuring respectful maternity care (RMC) during childbirth in India (face-to-face).

Multimedia Appendix 2 Summary of validity and reliability assessments by survey tool.

## Abbreviations

ANC: antenatal care
CATI: computer-assisted telephone interview
IVR: interactive voice response
LMIC: low- and middle-income country
MMR: maternal mortality ratio
MOHFW: Ministry of Health and Family Welfare
MP: Madhya Pradesh
RDD: Random Digit Dialing
RMC: respectful maternity care
SMS: short message service
USSD: Unstructured Supplementary Services Data
WHO: World Health Organization
