# Supplementary material for: Development of a Phone Survey Tool to Measure Respectful Maternity Care During Pregnancy and Childbirth in India: Study Protocol
Source: JMIR Res Protoc. 2019 Apr 25;8(4):e12173. doi: 10.2196/12173 (PMC6658236; doi:10.2196/12173)
Supplement: Multimedia Appendix 1 [file resprot_v8i4e12173_app1.pdf]

Appendix Table 1. Draft survey questions for measuring respectful maternity care (RMC) during childbirth in India (face-to-face). [a]

Domains	Question	Response option
**1.0 Physical or sexual abuse**		
**Use of force**		
	At any point during your stay for this delivery were you physically harmed by any of the health care workers? For example, physical abuse might include being hit, pinched, pulled or slapped.	1-Yes, 2-No, 98-Don't know
	What exactly happened?	(Prompted) 1-Kicked, 2-Pinched, 3-Slapped, 4-Pushed, 5-Beaten, 6-Raped, 7-Tied to delivery bed/ delivery coach, 97-Other, 98-Don't know
	Do you think that the poor treatment you described was influenced by any of the following? Please tell us the most important.	(Prompted) 1-Your social class, 2-Lack of insurance, 3-Your caste, 4-Your sex, 5-Your language, 6-Your religion, 7-Your political beliefs or other beliefs, 8-Your health status, 9-Lack of money or wealth, 10-Age, 97-Other specify, 11-Not applicable, 98-Don't know, 99-No response
**2.0 Verbal abuse**		
**Harsh or rude language**		
	At any point during your stay for this delivery did any health care provider talk or behave rudely?	1-Yes, 2-No, 98-Don't know
	What exactly happened?	(Prompted) 1-Shouted, 2-Scolded, 3-Threatened to withhold services, 4-Threatened with going to operation theatre, 5-Called by insulting name, 6-Laughed at or scorned, 7-Looked in a derogatory way, 97-other _____, 98-Don't know
**3.0 Stigma and discrimination**		
**Discrimination**		
	Do you think that the poor treatment you described was influenced by any of the following? Please tell us the most important.	(Prompted) 1-Your social class, 2-Lack of insurance, 3-Your caste, 4-Your sex, 5-Your language, 6-Your religion, 7-Your political beliefs or other beliefs, 8-Your health status, 9-Lack of money or wealth, 10-Age, 97-Other specify, 11-Not applicable, 98-Don't know, 99-No response
	All health workers treat patients equally	6- Strongly Agree<br>5-Agree<br>4- Somewhat Agree<br>3- Disagree<br>2- Somewhat Disagree<br>1- Strongly Disagree
**4.0 Failure to meet professional standards of care**		
**4.1 Refusal to provide pain relief**		
	Do you feel the doctors or nurses did everything they could to help control your pain?	6- Strongly Agree<br>5-Agree<br>4- Somewhat Agree<br>3- Disagree<br>2- Somewhat Disagree<br>1- Strongly Disagree
**4.2 Lack of informed consent process**		
	Did the doctors and nurses explain to you why they were doing examinations or procedures on you?	1-Yes, 2-No, 98-Don't know

Did the doctors, nurses or other staff at the facility ask your permission/consent before doing procedures and examinations on you?	1-Yes, 2-No, 98-Don't know
Please indicate if any of the following procedures were done without your permission (READ EACH OPTION AND CIRCLE RESPONSE)	(Prompted) 1-No 1, 2-Yes Tubal Ligation, 3-Yes Abdominal palpation, 4-Yes Vaginal examination, 5-Yes episiotomy, 97-Other specify, 98-Don't know
During the delivery, do you feel like you were able to be in the position of your choice?	1-Yes, 2-No, 98-Don't know
**4.3 Breaches of confidentiality**	
During your hospital stay, did health providers ever discuss your personal private health information in a way that others could hear?	1-Yes 2-No 98-Don't know
At any point during your stay for this delivery were you left un attended by health providers when you needed care?	1-Yes, 2-No, 98-Don't know
When were you left unattended?	(Prompted) 1-While in labor, 2-While delivering, 3-While experiencing a complication, 4-After delivery, 5-Baby after delivery, 97-Other specify, 98-Don't know
**4.4 Neglect, abandonment, or long delays**	
At any point during your stay for this delivery were you left un attended by health providers when you needed care?	1-Yes, 2-No, 98-Don't know
When were you left unattended?	(Prompted) 1-While in labor, 2-While delivering, 3-While experiencing a complication, 4-After delivery, 5-Baby after delivery, 97-Other specify, 98-Don't know
**5.0 Poor rapport between women and providers**	
**5.1 Poor communication**	
During your time in the health facility did the doctors, nurses, or other health care providers introduce themselves to you when they first came to see you?	1-yes, 2-no, 9898-don't know
Did you feel you could ask the doctors, nurses or other staff at the facility any questions you had?	6- Strongly Agree<br>5-Agree<br>4- Somewhat Agree<br>3- Disagree<br>2- Somewhat Disagree<br>1- Strongly Disagree
When you needed help, did you feel the doctors, nurses or other staff at the facility paid attention?	6- Strongly Agree<br>5-Agree<br>4- Somewhat Agree<br>3- Disagree<br>2- Somewhat Disagree<br>1- Strongly Disagree
**5.2 Lack of supportive care from health workers**	
How would you rate the respect the health workers showed you at this facility for this delivery? By respect I mean being treated with the care and attention you deserve	1-Excellent, 2-Very good, 3-Good, 4-Fair, 5-Poor, 98-Don't know
Were you allowed/ offered to drink liquids or eat any food while you were in labor?	1-Yes, 2-No, 98-Don't know
**5.3 Denial or lack of birth companions during labor and delivery**	

Were you allowed to have someone you wanted (outside of staff at the facility, such as family or friends) to stay with you during labor?	1-Yes 2-No 98-Don't know
Who was your companion during labor at the health facility?	(Prompted) 1-Father/Mother, 2-Mother in law, 3-Father in law, 4-Husband, 5-Child, 6-Other
Were you allowed to have someone you wanted to stay with you during delivery?	1-yes 2-No 98-Don't know
Who was your companion during delivery at the health facility?	(Prompted) 1-Father/Mother, 2-Mother in law, 3-Father in law, 4-Husband, 5-Child, 6-Other
**5.4 Lack of respect for women's preferred birth positions/ freedom of movement**	
Were you free to choose a position that was comfortable to you during your childbirth?	1-Yes 2-No 98-Don't know
Were you allowed to get up and walk around while you were in labor?	1-Yes 2-No 98-Don't know
**5.5 Detainment in facilities**	
At any point during your stay for this delivery were you or your baby prevented from leaving this facility because you could not pay?	1-Yes, 2-No, 98-Don't know, 99-No response/ refusal to answer
How long after delivery did you or your baby have to stay because of your inability to pay?	_____hours/ _____Days
**6.0 Health system conditions and constraints**	
**6.1 Lack of privacy**	
At any point during your delivery stay, were you covered up with a cloth or blanket or screened with a curtain so that you did not feel exposed?	1-Yes 2-No 98-Don't know
**6.2 Bribery and extortion**	
Were you or your family asked for a bribe or informal payment?	1-Yes 2-No 98-Don't know
**6.3 Physical condition of facilities**	
Was there water in the facility?	1- Yes, 2-No
Was there electricity in the facility?	1- Yes, 2-No
Thinking about the wards, washrooms and the general environment of the health facility, will you say the facility was very clean, clean, dirty, or very dirty?	6- Strongly Agree<br>5-Agree<br>4- Somewhat Agree<br>3- Disagree<br>2- Somewhat Disagree<br>1- Strongly Disagree
**6.0 Other questions**	
Overall, how satisfied are you with your experience during this delivery?	1-Very satisfied, 2-Somewhat satisfied, 3-Somewhat dissatisfied, 4-Very dissatisfied, 98-Don't Know
If you had more children in the future, where would like to deliver your next child?	1-At home, 2-Same health facility as recent delivery, 3-Different health facility than recent delivery, 98-Don't Know
Would you recommend the place of your most recent delivery to other women for their delivery?	6- Strongly Agree<br>5-Agree<br>4- Somewhat Agree<br>3- Disagree<br>2- Somewhat Disagree<br>1- Strongly Disagree

[a]Respondent demographic and socioeconomic characteristic questions not included in the above table
