# Supplementary material for: Development of a Phone Survey Tool to Measure Respectful Maternity Care During Pregnancy and Childbirth in India: Study Protocol
Source: JMIR Res Protoc. 2019 Apr 25;8(4):e12173. doi: 10.2196/12173 (PMC6658236; doi:10.2196/12173)
Supplement: Multimedia Appendix 2 [file resprot_v8i4e12173_app2.pdf]

Appendix Table 2. Summary of validity and reliability assessments by survey tool.

		Validity				Reliability				Response rates
		Content	Construct	Criterion	Longitudinal (responsiveness)	Test- retest (Agreement)	Internal consistency	Intermodal reliability	Floor and ceiling effects	Content and Length
ANC recipients	Survey 1. Face-to-face survey of RMC during ANC	√	√	√	√	√	√	√	√	√
Intra-partum	Survey 3. Face-to-face survey of RMC during childbirth	√	√	√	√	√	√	√	√	√
	Survey 4. RMC Phone-survey	√	√	√	√	√	√	√	√	√
